# Supplementary material for: A New Approach to The Synthesis of Polylactide/Polyacrylonitrile Block Copolymers
Source: Polymers (Basel). 2022 Apr 9;14(8):1529. doi: 10.3390/polym14081529 (PMC9031765; doi:10.3390/polym14081529)
Supplement: Supplementary file 1 [file polymers-14-01529-s001.zip › polymers-1642761-supplementary.pdf]

polymers

MDPI

Supplementary Materials: A New Approach to The Synthesis of Polylactide/Polyacrylonitrile Block Copolymers

Mateusz Grabowski, Bartłomiej Kost, Przemysław Kubisa and Melania Bednarek

1. Benzophenone Derivative Containing Primary -OH Group (HBP-ET)

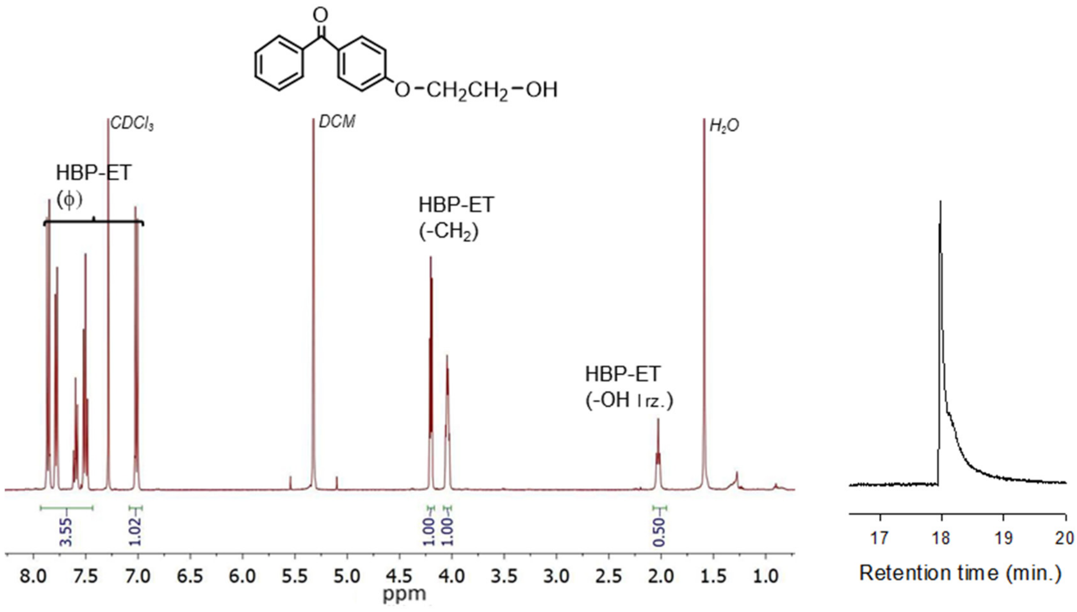

Figure S1. ^1H NMR spectrum ($CDCl_3$) and GC chromatogram for the synthesized HBP-ET.

2. PLAs Obtained by the Application Of HBP-ET as the Lactide Polymerization Initiator

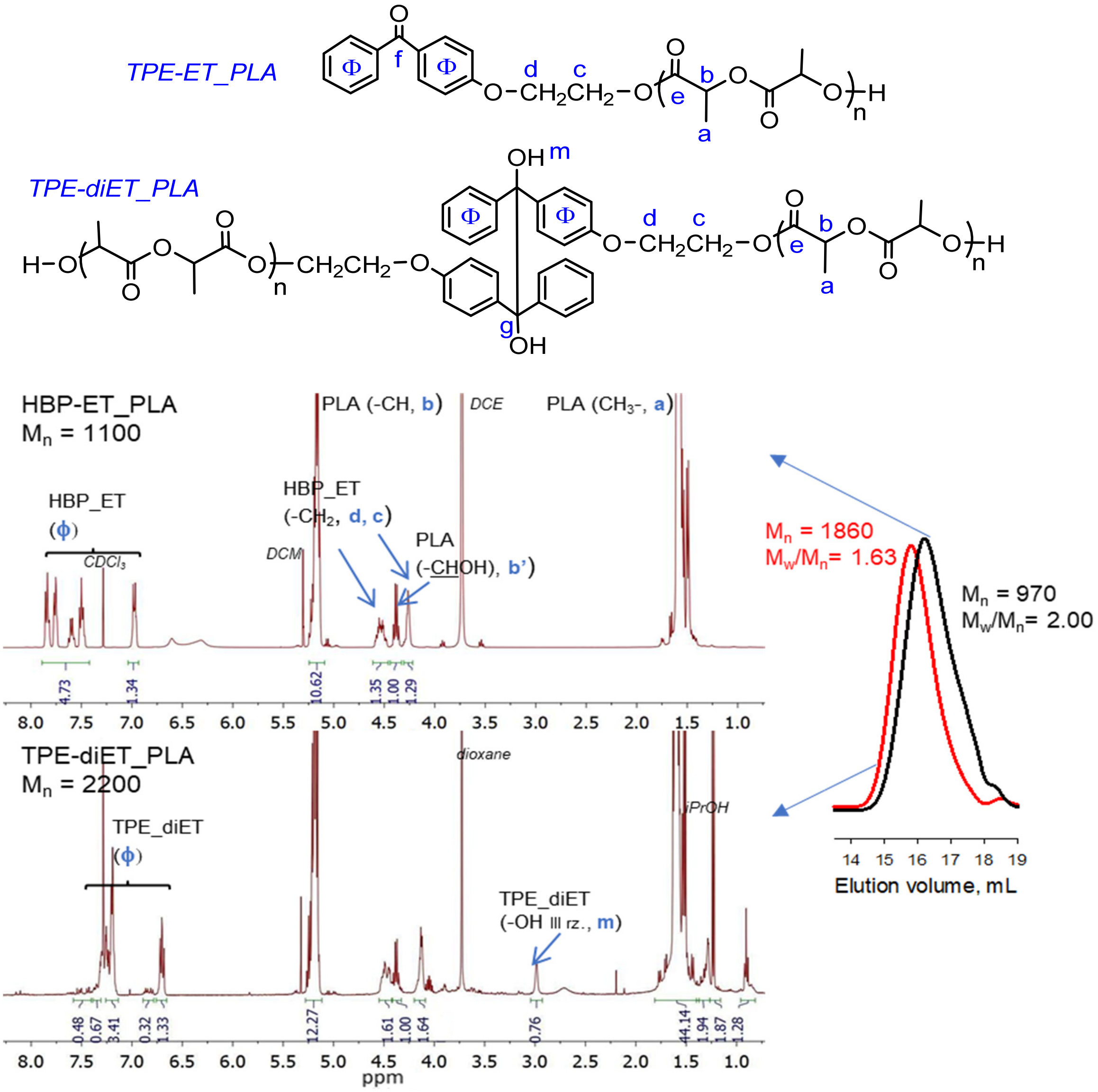

Figure S2. ^{1}H NMR spectra ($CDCl_3$) for HBP-ET_PLA, M_n=1100 and TPE-diET_PLA after coupling under UV radiation, M_n = 2200; PLAs formulas on the top.

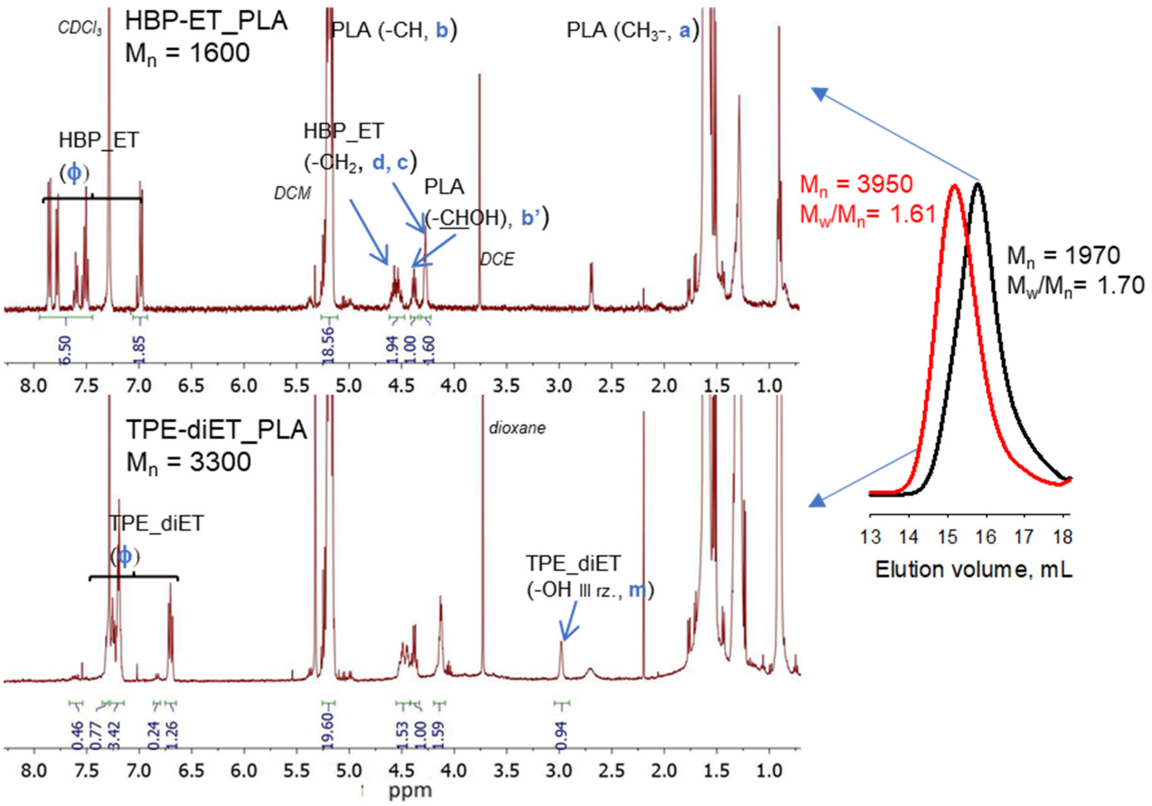

Figure S3. ^{1}H NMR spectra ($CDCl_3$) for HBP-ET_PLA, M_n=1600 and TPE-diET_PLA after coupling under UV irradiation, M_n = 3300.

Molecular weights of HBP-ET_PLA and TPE-diET_PLA polymers were calculated by the comparison of the intensities of signals corresponding to -<u>CH</u>(CH_3)-OH end groups at 4.3 ppm and corresponding to -<u>CH</u>(CH_3)- groups from PLA backbone at 5.2 ppm.

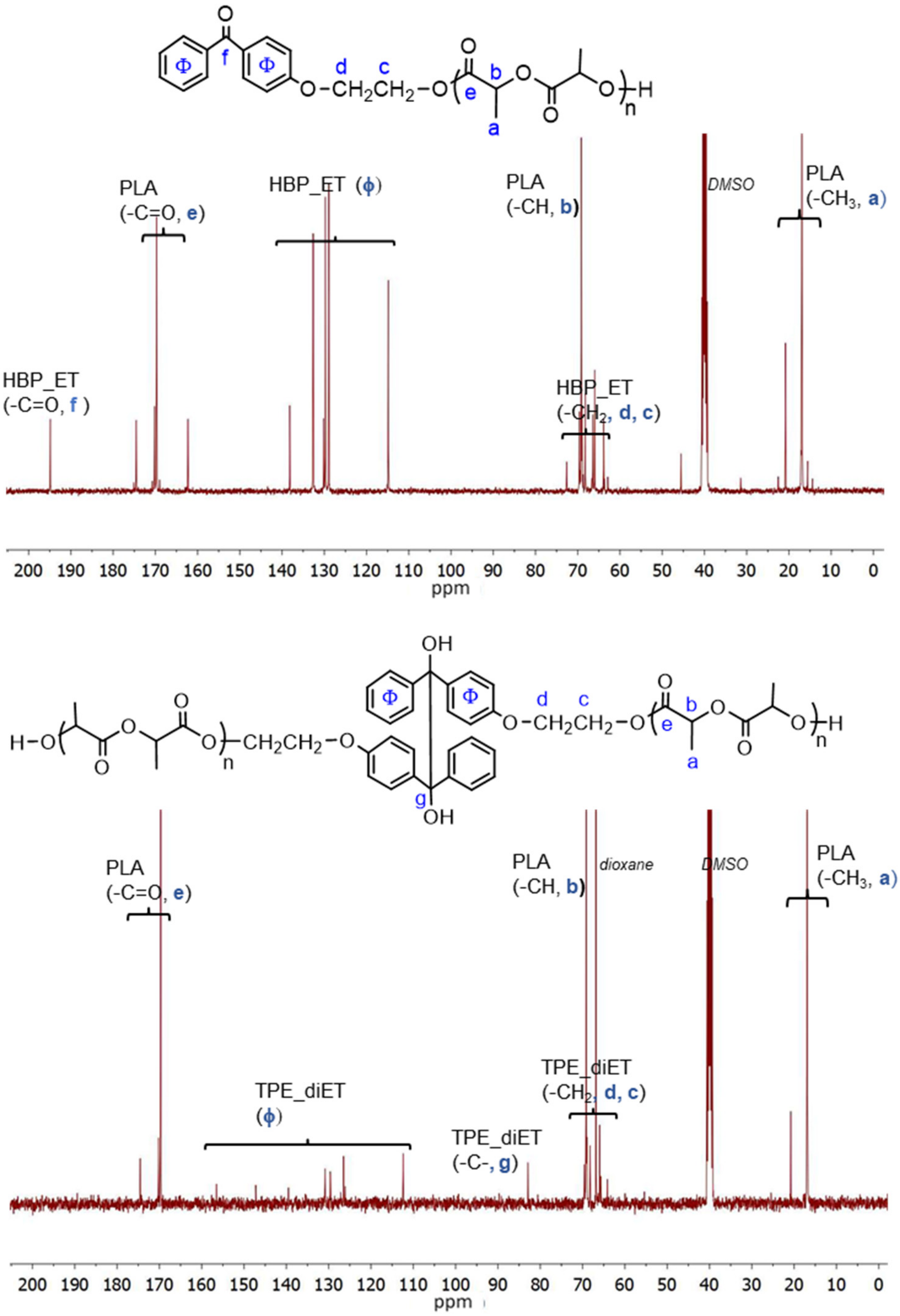

Figure S4. ^{13}C NMR spectra for HBP-ET_PLA (M_n = 700) and TPE-diET_PLA (M_n = 1400) after the coupling reaction. The signal characteristic for carbonyl group of benzophenone moiety present in HBP-ET_PLA at ~195 ppm disappears after coupling and a new signal at ~82 ppm corresponding to quaternary carbon atom in TPE unit appears.

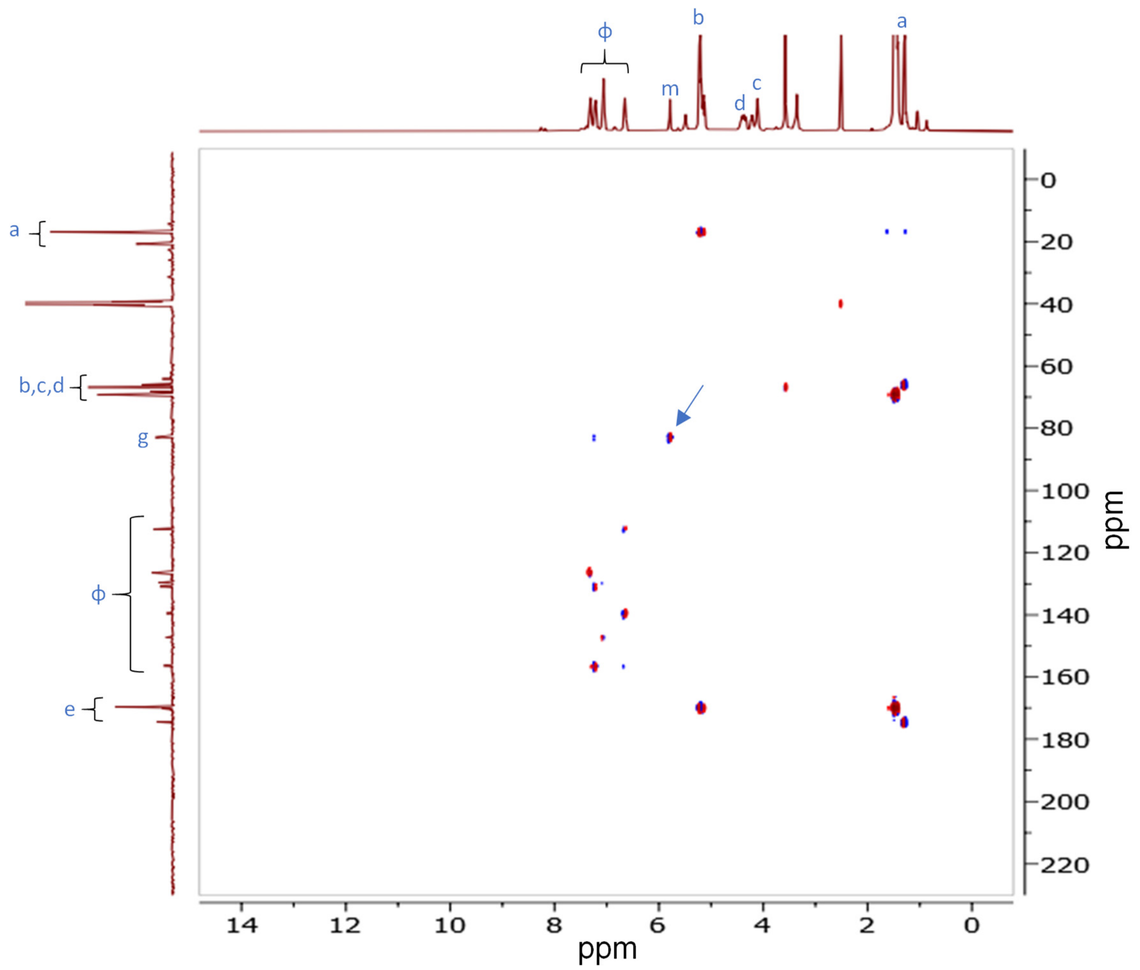

Figure S5. $^{1}H^{13}C$-HMBC NMR spectra for TPE-diET_PLA (M_n = 1400). An arrow indicates correlation between quaternary carbon atom and tertiary -OH group from TPE-diET unit.

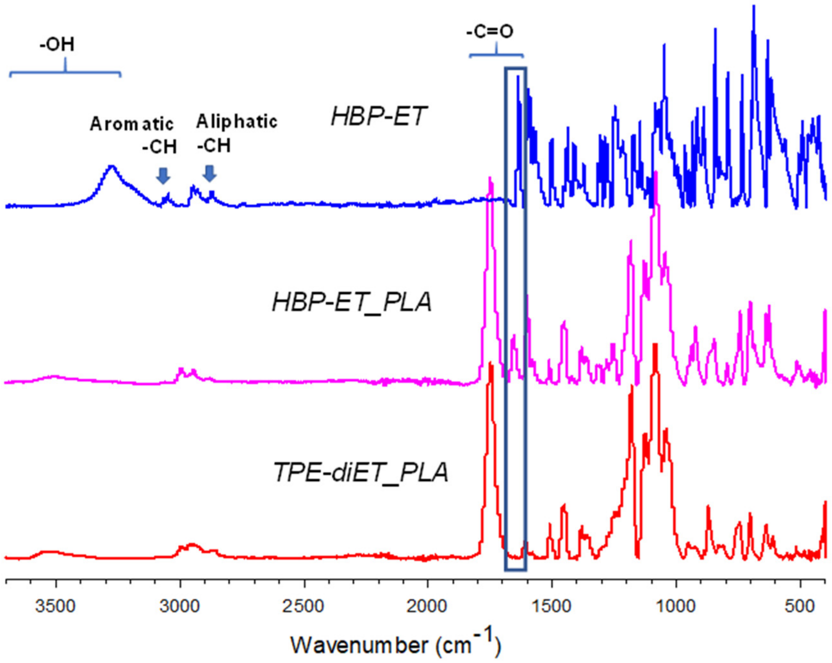

Figure S6. FTIR spectra for HBP-ET initiator, HBP-ET_PLA polymer (M_n = 700) and TPE-diET_PLA after coupling of HBP-ET_PLA (M_n = 1400).

It is visible that the signal corresponding to carbonyl group in HBP-ET at ~1650 cm^{-1} disappeared after coupling of HBP-ET_PLA leading to TPE-diET_PLA.

3. Conversion of Acrylonitrile for Performed Copolymer Syntheses and the Kinetic Experiment

Conversion of AN was calculated by the comparison of the intensities of signals corresponding to unsaturated groups at 5.8-6.4 ppm from AN monomer (3 protons) with the intensity of the well separated signal corresponding to methylene protons from PAN chain at 2.0-2.25 ppm (2 protons).

Decomposition percent of TPE groups (kinetic experiment) was calculated by the comparison of the intensities of signals in the range 6.6–7.8 ppm which remained after abstraction of signals at 6.65, 7.06, 7.25, and 7.36 ppm corresponding to initial TPE group, with the sum of intensities of all signals in the range 6.6–7.8 ppm.

polymers

4. Analysis of Acrylonitrile Polymerization Products

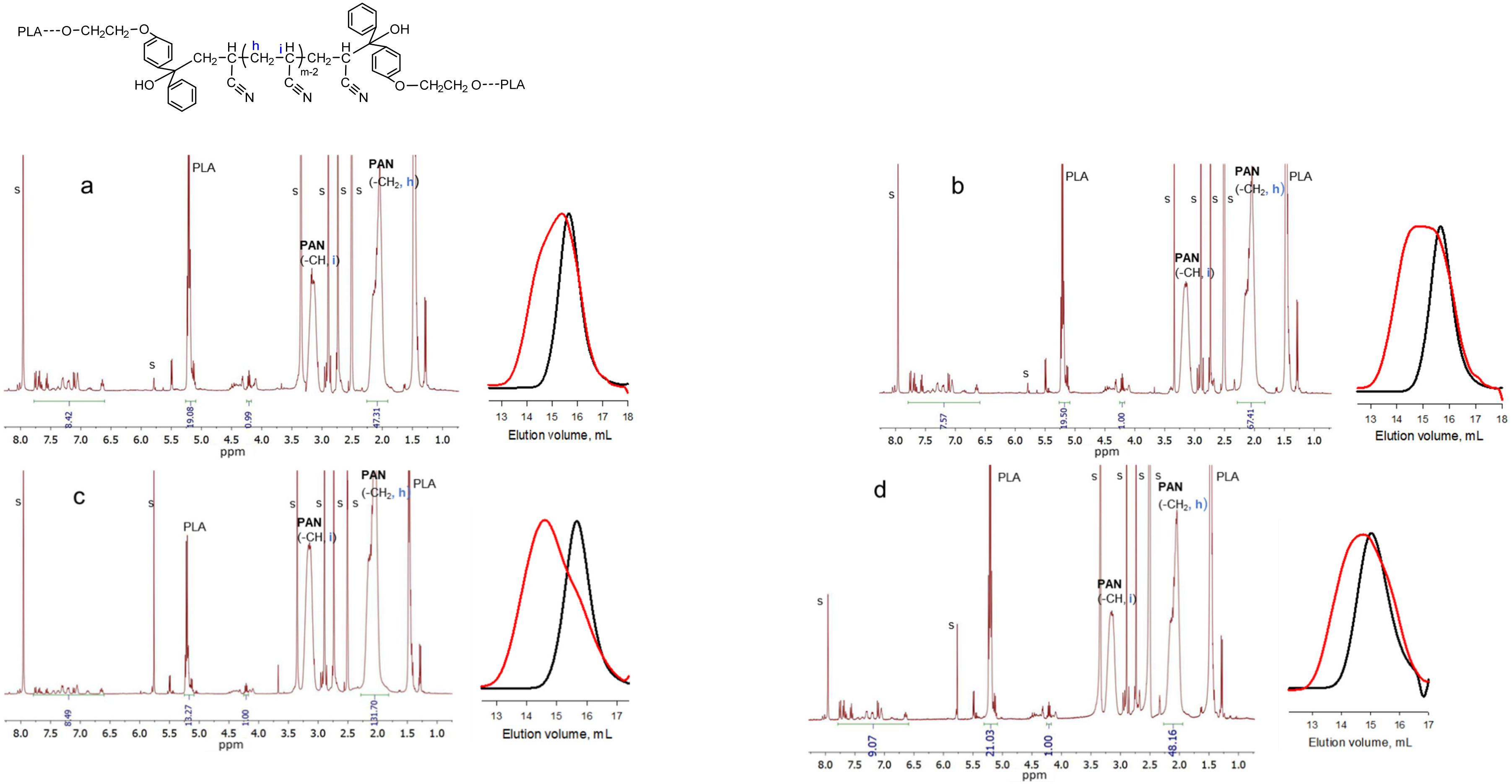

Figure S7. ^{1}H NMR spectra (DMSO-d6) and SEC curves (red line) for the products of reactions No: (**a**) 2, (**b**) 3, (**c**) 4, (**d**) 5 in Table 1 in the main text; "s" denotes solvent, assignments of other signals can be found in Fig. 4 in the main text; black SEC line corresponds to starting TPE-diET_PLA; The expected structure of PLA-PAN-PLA copolymers on the top.

Molecular weights of TPE-diET_PLA/PAN copolymers were calculated as follows:

1. $M_{PLA\ block}$ = $P_{n\ PLA}$ (intensity of the signal corresponding to -<u>CH</u>(CH_3)- groups from PLA backbone at 5.2 ppm / intensity of the signal corresponding to -<u>CH</u>(CH_3)-OH end groups at 4.3 ppm) × 144
2. $M_{PAN\ block}$ = $P_{n\ PAN}$ ($P_{n\ PLA}$ x AN/PLA ratio from Table 1) × 53
3. M_n of copolymer = $M_{PLA\ block}$ + $M_{PAN\ block}$ + 486 ($M_{TPE\text{-}diET}$)
